
JOURNAL INFORMATION
==============================
NLM Title Abbreviation: Wirtschaftsdienst
Journal ID: Wirtschaftsdienst
NLM Journal ID: 101086612

Wirtschaftsdienst (Hamburg, Germany : 1949)

ISSN: 0043-6275

ARTICLE INFORMATION
==============================
PMCID: PMC7678250
PMID: 33250539
DOI: 10.1007/s10273-020-2788-y
Article ID: 2788
Article version: 1
Subjects: Ökonomische Trends

Die Corona-Krise kommt mit Wucht zurück

Clemens Marius MClemens@diw.de

Dany-Knedlik Geraldine gdanyknedlik@diw.de

Junker Simon sjunker@diw.de

Michelsen Claus cmichelsen@diw.de

grid.8465.f 0000 0001 1931 3152 Konjunkturpolitik, DIW Berlin, Mohrenstr. 58, 10117 Berlin, Deutschland
Electronic publication date: 2020 Nov 20
Print publication date: 2020
Volume: 100
Issue: 11
First page: 896
Last page: 898
Copyright: © Der/die Autor(en) 2020
License: Open Access: Dieser Artikel wird unter der Creative Commons Namensnennung 4.0 International Lizenz (https://creativecommons.org/licenses/by/4.0/deed.de) veröffentlicht.
Open Access wird durch die ZBW - Leibniz-Informationszentrum Wirtschaft gefördert.
License URL: https://creativecommons.org/licenses/by/4.0/

issue-copyright-statement: © The Author(s) 2020

==============================

Rekordwachstum im 3. Quartal des Jahres 2020, niedrige Infektionszahlen und praktisch kaum Auswirkungen der Krise auf den Arbeitsmärkten in Europa: Für viele hat die Corona-Krise in den Sommermonaten dieses Jahres ihren Schrecken verloren. Fast vergessen schien der heftigste Einbruch der Wirtschaftsleistung innerhalb eines Quartals seit dem Zweiten Weltkrieg. Und so revidierten viele Prognostiker*innen ihre Einschätzungen über die wirtschaftliche Entwicklung kräftig nach oben (Holtemöller et al., 2020) – der Ausblick hellte sich nicht nur in Deutschland bis Anfang Oktober 2020 deutlich auf. Mit zunehmender wirtschaftlicher Aktivität stellte sich auch ein Stück weit eine Normalisierung des gesellschaftlichen Lebens ein: Schulen und Kindergärten im Regelbetrieb, geöffnete Schwimmbäder, Zuschauer*innen in den Fußballstadien und gut gefüllte Restaurants machten offenbar leichtsinnig. Denn so schön die Sommermonate auch waren: Das Infektionsrisiko blieb bestehen.

Mit Beginn der kälteren Jahreszeit stiegen die Infektionszahlen zunächst allmählich, dann aber mit zunehmender Geschwindigkeit. Schon Mitte September 2020, zum Ende der Schulferien, wurden wieder mehr als 2.000 Corona-Fälle pro Tag gemeldet, vor allem in der Bevölkerungsgruppe zwischen 15 und 34 Jahren. Dies mag auch erklären, dass trotz dieser Zahlen kaum Dramatisches aus dem Gesundheitswesen berichtet wurde. Allerdings war eine Ausbreitung der Infektionen in andere Bevölkerungsgruppen nur eine Frage der Zeit. Verschiedene Untersuchungen haben gezeigt, dass die räumliche Dynamik der Verbreitung stark durch das Pendelgeschehen bestimmt wird und die Intensität der Ansteckungen auch von der Witterung abhängig ist (beispielsweise Mense und Michelsen, 2020). Und so stiegen die Infektionszahlen im Oktober auf immer neue Rekordhöhen. Zuletzt wurden mehr als 20.000 Neuinfektionen an einem Tag gezählt – gut das Dreifache dessen, was in der Spitze im Frühjahr in Deutschland erfasst wurde.[1] Mittlerweile sind die Gesundheitsämter vielfach nicht mehr in der Lage, das Infektionsgeschehen nachzuvollziehen und Infektionsketten wirksam zu unterbrechen. Drei Viertel aller Infektionen können nach Angaben des Robert-Koch-Instituts nicht mehr auf ihren Ursprung zurückgeführt werden.

Lange haben sich die Bundesregierung und die Landesregierungen schwer damit getan, auf diese Entwicklung zu reagieren. Appelle und Beteuerungen, alles gegen eine neue Verbreitung des Virus tun zu wollen, haben aber keinen Abbruch des Infektionsgeschehens bewirkt. Und so blieb angesichts dessen enormer Geschwindigkeit kaum eine andere Wahl, als erneut weitreichende Beschränkungen des gesellschaftlichen Lebens und der Geschäftstätigkeit einiger Wirtschaftsbereiche mit besonders schwer nachvollziehbaren sozialen Kontakten zu beschließen. Am 2. November 2020 mussten Theater und Museen, Freizeiteinrichtungen, Restaurants, Bars und Hotels und zahlreiche Dienstleistungen erneut in den Lockdown. Ziel ist es, die Kontakte aller Menschen im Land für vier Wochen um 75 % zu reduzieren, ohne Schulen, Kitas und den Einzelhandel schließen zu müssen. Aufgefordert wird zum Arbeiten von zuhause und zum Verzicht auf persönliche Begegnungen außerhalb der Familie (Deutscher Bundestag, 2020). Deutschland ist allerdings längst nicht das einzige Land, das erneut in den Lockdown geht. Die meisten europäischen Länder haben weitreichende Beschränkungen beschlossen.

## Zweite Welle und Lockdown kosten 19 Mrd. Euro an Wirtschaftsleistung in Deutschland

Die zweite Infektionswelle hat Folgen für die deutsche Wirtschaft: Getroffen werden in erster Linie Dienstleistungsbereiche wie das Gastgewerbe: Nachdem sich unter anderem Restaurants, Kneipen und Hotels auch durch die zügige Erstellung und Umsetzung von Hygienekon-

1 Die Positivraten der Corona-Tests im Frühjahr deuten allerdings darauf hin, dass die Dunkelziffer damals weitaus größer war. Dennoch steigen auch jetzt die Positivraten erheblich an, was eine hohe Infektionsdynamik in der Bevölkerung nahelegt (Dorn et al., 2020).

zepten im Sommer wieder nach oben getastet hatten, brechen die Umsätze jetzt ein zweites Mal in diesem Jahr nahezu vollständig ein. Erste Schätzungen des DIW Berlin legen nahe, dass sich die Wertschöpfung im Durchschnitt des 4. Quartals in diesen Bereichen halbieren dürfte, wobei sich hier der vergleichsweise ertragsstarke Oktober und – sofern der Lockdown Ende November 2020 endet – eine Erholung im Dezember positiv auswirken.

Ähnlich wird die Entwicklung auch in den übrigen besonders betroffenen Branchen verlaufen. In diese fallen etwa Tätigkeiten aus den Bereichen Kultur und Sport, die im November erneut untersagt sind. Der Rückgang wird dabei nicht so stark ausfallen wie im Frühjahr, was aber einzig daran liegen dürfte, dass er von einem bis zuletzt noch immer recht niedrigem Niveau aus erfolgt. Im Vergleich zum Frühjahr bleibt der Einzelhandel wohl eher verschont. Die Geschäfte dürfen im November mit Einschränkungen geöffnet bleiben. Die Erfahrungen aus dem Frühjahr zeigen aber, dass die Kauflaune durch das Infektionsgeschehen deutlich gedämpft wird. Ohnehin haben sich infolge der Schließungen im Frühjahr große Teile des Marktgeschehens ins Internet verlagert. Abgesehen von den Effekten für den Großhandel dürften im Handelssegment größere Einbrüche ausbleiben.

Alles in allem wird die zweite Infektionswelle weniger gravierende Wirtschaftseinbußen mit sich bringen als im Frühjahr. Dennoch überlagert der neuerliche Einbruch nun den kräftigen Aufholprozess der deutschen Wirtschaft, der sich in einem Rekordzuwachs im 3. Quartal niedergeschlagen hat. Die Einschränkungen im November dämpfen das Wachstum der deutschen Wirtschaft im 4. Quartal um 2,5 Prozentpunkte oder rund 19 Mrd. Euro. So wird der im Mai 2020 begonnene Erholungsprozess unterbrochen. Mit einem Rückgang des Bruttoinlandsprodukts um 1 % gegenüber dem Vorquartal dürfte Deutschland zurück in die Rezession fallen (Clemens et al., 2020).

Ein Risiko liegt in der Entwicklung der Industrie, die bei den unterstellten eher geringen Rückschlägen auf den internationalen Absatzmärkten in den vorliegenden Berechnungen nur vergleichsweise milde ausgebremst wird, zumal sie von den Maßnahmen des Lockdowns nicht direkt betroffen ist. Mit Blick auf die in vielen Ländern aktuell ergriffenen Maßnahmen kann der Außenhandel und damit die deutsche Exportindustrie aber durchaus deutlicher einbrechen als in den vorliegenden Berechnungen unterstellt.

Durch die Unterbindung von sozialen Aktivitäten wird vor allem der private Verbrauch beeinträchtigt (vgl. Tabelle 1). Angesichts erneuter Umsatzeinbußen werden auch investive Ausgaben zurückgestellt. Verstärkt wird die Investitionszurückhaltung durch die gestiegene Unsicherheit

Tabelle 1

### Effekte der zweiten Welle und eines Lockdowns

Quartalsdaten zur Entwicklung der Verwendungs- und Entstehungskomponenten des realen Bruttoinlandsprodukts, Veränderung gegenüber dem Vorquartal in %; saison- und kalenderbereinigt

| | 2020 in % | | | | Effekt des Lockdowns | |
|---|---|---|---|---|---|---|
| | I | II | III | IV[a] | % | Mrd. Euro |
| Privater Verbrauch | -2,5 | -10,9 | 8,9 | -1,4 | -3,6 | -14,7 |
| Öffentliche Konsumausgaben | 0,6 | 1,5 | 0,8 | 1,0 | 0 | 0 |
| Investitionen | | | | | | |
| Bauten | 5,1 | -4,2 | -1,3 | 0,8 | 0 | 0 |
| Ausrüstungen | -7,3 | -19,6 | 15,0 | -1,0 | -4,3 | -2,1 |
| Sonstige Investitionen | -4,1 | 0,6 | 4,0 | 0,2 | -0,4 | -0,1 |
| Exporte | -3,3 | -20,3 | 16,5 | -2,4 | -5,0 | -17,8 |
| Importe | -1,9 | -16,0 | 10,8 | -1,3 | -4,7 | -15,0 |
| Bruttoinlandsprodukt | -2 | -9,7 | 8,2 | -1,0 | -2,5 | -19,3 |
| Bruttowertschöpfung | -1,7 | -9,9 | 8,2 | -1,0 | -2,5 | -17,7 |
| Verarbeitendes Gewerbe | -4,0 | -16,1 | 14,0 | -1,0 | -3,6 | -5,2 |
| Baugewerbe | 4,6 | -3,8 | -1,5 | 0,8 | 0 | 0 |
| Handel, Gastgewerbe, Verkehr | -1,4 | -12,4 | 15,6 | -5,1 | -6,9 | -8,2 |
| Unternehmensdienstleistung | -2,2 | -14,3 | 6,5 | -0,5 | -2,0 | -1,5 |
| Öffentliche Dienstleistung, Erziehung, Gesundheit | -1,2 | -8,8 | 8,5 | 1,4 | 0 | 0 |
| Sonstige Dienstleistung (Kultur, Sport, Unterhaltung) | -2,9 | -17,6 | 12,0 | -7,3 | -8,4 | -2,1 |

[a] Prognose.

Quelle: Statistisches Bundesamt, Berechnungen des DIW Berlin.

über die weltweiten Auswirkungen der Pandemie. Dies macht sich auch im Außenhandel bemerkbar, wenngleich weniger als im Frühjahr.

## Folgen auf dem Arbeitsmarkt sichtbar

Auch im zweiten Lockdown dürften die Unternehmen den Produktionsausfall sofern möglich durch Kurzarbeit auffangen. Viele Unternehmen sind durch die lange Durststrecke jedoch angeschlagen, entsprechend fällt der Beschäftigungsabbau im Verhältnis wohl etwas stärker aus. Die Zahl der Erwerbstätigen dürfte im 4. Quartal angesichts des geringeren Wirtschaftseinbruchs als im Frühjahr, mit etwa 100.000 Personen nicht so gravierend sinken. Mit deutlicheren Anstiegen ist bei der Zahl der Kurzarbeiter*innen zu rechnen: Diese könnte um 400.000 nach oben schnellen.

Tabelle 2
**Arbeitsmarkteffekte**
in 1.000

| | 2020 | | | |
|---|---|---|---|---|
| | I | II | III | IV[a] |
| Erwerbstätige | 45.319 | 44.692 | 44.644 | 44.555 |
| Änderung | -13 | -627 | -48 | -89 |
| Kurzarbeitende | 949 | 5.369 | 2.776 | 3.170 |
| Änderung | 844 | 4.420 | -2.592 | 393 |
| Arbeitslose | 2.269 | 2.816 | 2.909 | 2.957 |
| Änderung | -11 | 547 | 3 | 47 |

[a] Prognose.

Quellen: Statistisches Bundesamt; Bundesagentur für Arbeit; Berechnungen des DIW Berlin.

Die Arbeitslosigkeit wird wohl spiegelbildlich zum Beschäftigungsabbau steigen, wenn auch nicht im gleichen Maße. Im April hatte auch eine Rolle gespielt, dass Menschen, die sich zu Beginn des Lockdowns in arbeitsmarktpolitischen Maßnahmen befunden hatten, stattdessen als arbeitslos registriert wurden. Dies kann auch ab November die Arbeitslosenzahlen noch höher ausfallen lassen. Unter diesen Vorzeichen dürfte die Zahl der Arbeitslosen im 4. Quartal um rund 50.000 Personen steigen (vgl. Tabelle 2).

## Bundesregierung federt wirtschaftlichen Aufprall ab

Die zweite Infektionswelle und Verschärfungen der Infektionsschutzmaßnahmen in Deutschland sowie in anderen großen europäischen Ländern kosten die deutsche Wirtschaft in diesem Jahr gut 19 Mrd. Euro und schmerzen insbesondere jene Branchen, die ohnehin schon stark unter der Krise gelitten haben. Dazu zählen vor allem das Gastgewerbe sowie der Bereich Kultur und Veranstaltungen. Die neuerlichen Einschränkungen dürften dennoch das kleinere Übel sein. Wenn es gelingt, die zweite Corona-Infektionswelle zu stoppen oder zumindest signifikant abzuflachen, ist nicht nur der Gesundheit der Bevölkerung gedient, sondern auch den Interessen der Wirtschaft. Beides geht Hand in Hand und ist die Voraussetzung dafür, dass die deutsche Wirtschaft schon im kommenden Jahr wieder wachsen kann.

Die Bundesregierung hat bereits zusätzliche Maßnahmen veranlasst, um die wirtschaftlichen Folgen der zweiten Welle abzumildern: Direkt betroffene Unternehmen erhalten außerplanmäßige Wirtschaftshilfen. Diese sehen für Kleinst- und Kleinunternehmen eine einmalige Kostenerstattung in Höhe von 75 % des Umsatzes aus dem Vorjahresmonat vor, die mit in Anspruch genommenem Kurzarbeitergeld und den Überbrückungshilfen verrechnet wird. Solo-Selbstständige können wählen zwischen dem Umsatz des Vorjahresmonats und dem durchschnittlichen Umsatz des Jahres 2020, für größere Unternehmen werden Einzelfallregelungen getroffen. Diese Hilfe wird – Stand jetzt – einmalig und nur an direkt vom Lockdown betroffene Unternehmen gezahlt. Danach können zugangsberechtigte Unternehmen die bereits im Herbst verlängerten Kurzarbeitergeldregelungen und die Überbrückungshilfen in Anspruch nehmen.[2] Dies gilt auch für indirekt betroffene Unternehmen, sofern sie die Zugangskriterien erfüllen. Bisher sind Klein-, Kleinstfirmen und Soloselbstständige anspruchsberechtigt, wenn sie entweder zwischen April und August 2020 im Durchschnitt einen Umsatzrückgang von mindestens 30 % erwirtschaftet haben oder aber in zwei aufeinander folgenden Monaten des Zeitraums einen Umsatzrückgang von mindestens 50 %. Für die Überbrückungshilfen III sollen diese Konditionen gelockert werden. Zudem werden jetzt auch kleinere Unternehmen bei Liquiditätsengpässen auf das KfW-Schnellkreditprogramm zurückgreifen können, zu dem bisher nur Firmen mit mehr als 50 Personen Zugang hatten.

Auch das im Juni 2020 beschlossene Konjunkturprogramm stabilisiert. So sind investiv wirkende Ausgaben in Höhe von insgesamt rund 50 Mrd. Euro geplant. Ein Großteil dieser Mittel kann ab dem Jahr 2021 abgerufen werden und damit auch die negativen Auswirkungen der zweiten Welle verringern – allerdings nur, wenn die zweite Infektionswelle durch die teilweisen Schließungen gestoppt werden kann. Gelingt dies nicht, könnte der wirtschaftliche Schaden für Deutschland deutlich größer sein und höhere Mittel in Anspruch nehmen, denn viele private Unternehmen haben nicht mehr die finanziellen Rücklagen und die Widerstandsfähigkeit, die sie noch zu Anfang der ersten Infektionswelle im März und April 2020 hatten.

2 Der erleichterte Zugang zur Kurzarbeit und die Erhöhung des Kurzarbeitergeldes ab dem vierten Monat für Beschäftigte, die die Arbeitszeit um mindestens die Hälfte reduzieren, wurde bereits im Herbst bis Ende 2021 verlängert. Der Antragszeitraum für Überbrückungshilfen wurde im Zuge des partiellen Lockdowns ein weiteres Mal bis Mitte 2021 ausgeweitet.

Dr. Marius Clemens, Dr. Geraldine Dany-Knedlik und Dr. Simon Junker sind wissenschaftliche Mitarbeiter*nnen am Deutschen Institut für Wirtschaftsforschung (DIW Berlin).

Dr. Claus Michelsen leitet die Abteilung Konjunkturpolitik am DIW Berlin.


Literatur

Clemens M Dany-Knedlik G Junker und S Michelsen C Zweite Corona-Infektionswelle: Deutsche Wirtschaft wird zum Jahresende schrumpfen DIW aktuell 2020 55 5
Deutscher Bundestag (2020), Regierungserklä rung durch die Bundeskanzlerin zur Bewältigung der Covid-19-Pandemie, Stenografischer Bericht der 186. Sitzung des Bundestags, Plenarprotokoll, 19/186.
Dorn, F., C. Fuest, D. Gstrein, A. Peichl und M. Stöckli (2020), Corona-Infektionen und die Dunkelziffer: Vergleichen wir Äpfel mit Birnen?, ifo Schnelldienst Digital, 1(12).
Holtemöller O Kooths S Michelsen C Schmidt und T Wollmershäuser T Erholung verliert an Fahrt - Wirtschaft und Politik weiter im Zeichen der Pandemie Wirtschaftsdienst 2020 100 11 885 889
Mense A Michelsen C Räumliche Ausbreitung von COVID-19 durch interregionale Verflechtungen Wirtschaftsdienst 2020 100 6 416 421 10.1007/s10273-020-2674-7 32834156 PMC7335932
